# The Complete Chloroplast Genome Sequence of *Podocarpus lambertii*: Genome Structure, Evolutionary Aspects, Gene Content and SSR Detection

**DOI:** 10.1371/journal.pone.0090618

**Published:** 2014-03-04

**Authors:** Leila do Nascimento Vieira, Helisson Faoro, Marcelo Rogalski, Hugo Pacheco de Freitas Fraga, Rodrigo Luis Alves Cardoso, Emanuel Maltempi de Souza, Fábio de Oliveira Pedrosa, Rubens Onofre Nodari, Miguel Pedro Guerra

**Affiliations:** 1 Laboratório de Fisiologia do Desenvolvimento e Genética Vegetal, Programa de Pós-graduação em Recursos Genéticos Vegetais, Universidade Federal de Santa Catarina, Florianópolis, Santa Catarina, Brazil; 2 Departamento de Bioquímica e Biologia Molecular, Núcleo de Fixação Biológica de Nitrogênio, Universidade Federal do Paraná, Curitiba, Paraná, Brazil; 3 Departamento de Biologia Vegetal, Universidade Federal de Viçosa, Viçosa, Minas Gerais, Brazil; University of Minnesota, United States of America

## Abstract

**Background:**

*Podocarpus lambertii* (Podocarpaceae) is a native conifer from the Brazilian Atlantic Forest Biome, which is considered one of the 25 biodiversity hotspots in the world. The advancement of next-generation sequencing technologies has enabled the rapid acquisition of whole chloroplast (cp) genome sequences at low cost. Several studies have proven the potential of cp genomes as tools to understand enigmatic and basal phylogenetic relationships at different taxonomic levels, as well as further probe the structural and functional evolution of plants. In this work, we present the complete cp genome sequence of *P. lambertii*.

**Methodology/Principal Findings:**

The *P. lambertii* cp genome is 133,734 bp in length, and similar to other sequenced cupressophytes, it lacks one of the large inverted repeat regions (IR). It contains 118 unique genes and one duplicated tRNA (*trnN*-GUU), which occurs as an inverted repeat sequence. The *rps16* gene was not found, which was previously reported for the plastid genome of another Podocarpaceae (*Nageia nagi*) and Araucariaceae (*Agathis dammara*). Structurally, *P. lambertii* shows 4 inversions of a large DNA fragment ∼20,000 bp compared to the *Podocarpus totara* cp genome. These unexpected characteristics may be attributed to geographical distance and different adaptive needs. The *P. lambertii* cp genome presents a total of 28 tandem repeats and 156 SSRs, with homo- and dipolymers being the most common and tri-, tetra-, penta-, and hexapolymers occurring with less frequency.

**Conclusion:**

The complete cp genome sequence of *P. lambertii* revealed significant structural changes, even in species from the same genus. These results reinforce the apparently loss of rps16 gene in Podocarpaceae cp genome. In addition, several SSRs in the *P. lambertii* cp genome are likely intraspecific polymorphism sites, which may allow highly sensitive phylogeographic and population structure studies, as well as phylogenetic studies of species of this genus.

## Introduction

Extant gymnosperms are considered the most ancient group of seed-bearing plants that first appeared approximately 300 million years ago [1]. They consist of four major groups, including Gnetophytes, Conifers, Cycads and Ginkgo. Podocarpaceae are considered the most diverse family of Conifers, and much of this diversity has taken place within the *Podocarpus* and *Dacrydium* genera [2]. The Podocarpaceae family comprises 18 genera and 173 species distributed mainly in the Southern Hemisphere, but extending to the north in subtropical China, Japan, Mexico and the Caribbean [3], [4].

The *Podocarpus sensu lato* (*s.l.*) genus comprises nearly 100 species, widely spread throughout the Southern Hemisphere and northward to the West Indies, Mexico, southern China and southern Japan [5]. Ledru et al. [6] described that *Podocarpus* populations in Brazil are widely dispersed in eastern Brazil, from north to south, and three endemic species have been reported: *Podocarpus sellowii* Klotzch ex Endl, *Podocarpus lambertii* Klotzch ex Endl, and *Podocarpus brasiliensis* de Laubenfels [7]. *P. lambertii* is a native species from the Araucaria Forest, a subtropical moist forest ecoregion of the Atlantic Forest Biome, which is considered one of the 25 biodiversity hotspots of the world [8]. It is a dioecious evergreen tree of variable height, measuring 1–10 m, shade-tolerant, adapted to high frequency and density of undergrowth [9].

Phylogeny analyses by maximum parsimony of Podocarpaceae family using 18S rDNA gene sequencing and morphological characteristics indicated Podocarpaceae as monophyletic and *Podocarpus s.l.* and *Dacrydium s.l.* genera as unnatural [2]. This author concluded that single-gene studies rarely result in perfect phylogenies, but they could provide a basis for choosing between competing hypotheses. Parks et al. [10] suggested chloroplast (cp) genome sequencing as an efficient option for increasing phylogenetic resolution at lower taxonomic levels in plant phylogenetic and genetic population analyses.

The advancement of next-generation sequencing technologies has enabled the rapid acquisition of whole cp genome sequences at low cost when compared with traditional sequencing approaches. Chloroplast sequences are available for all families of Conifers: Cephalotaxaceae [11], Cupressaceae [12], Pinaceae [13]–[15], Podocarpaceae (NC_020361.1) and [16], Taxaceae (NC_020321.1), and Araucariaceae [16]. For *Podocarpus* genus, the cp sequence of only one species has recently been obtained: the endemic New Zealand *Podocarpus totara* G. Benn. ex Don (NC_020361.1).

Several studies have proven the potential of cp genomes as tools to understand enigmatic and basal phylogenetic relationships at different taxonomic levels, as well as probe the structural and functional evolution of plants [11], [17]–[20]. Hirao et al. [12] sequenced the cp genome of the first species in the Cupressaceae family, *Cryptomeria japonica*. They reported the deletion of one large inverted repeat (IR), numerous genomic rearrangements, and many differences in genomic structure between *C. japonica* and other land plants, thus supporting the theory that a pair of large IR can stabilize the cp genome against major structural rearrangements and, in turn, providing new insights into both the evolutionary lineage of coniferous species and the evolution of the cp genome [12], [21], [22].

Chloroplast genome sequencing in gymnosperms also brought insights into evolutionary aspects in Gnetophytes. Wu et al. [23] considered that the reduced cp genome size in Gnetophyte was based on a selection toward a lower-cost strategy by deletions of genes and noncoding sequences, leading to genomic compactness and accelerated substitution rates. More recently, comparative analysis of the cp genomes in cupressophytes and Pinaceae provided inferences about the loss of large IR [11], [20]. On one hand, Wu et al. [20] and Wu and Chaw [16] argue that each Pinaceae and cupressophyte lost a different copy of IR. On the other hand, Yi et al. [11] showed that distinct isomers are considered as alternative structures for the ancestral cp genome of cupressophyte and Pinaceae lineages. Therefore, it is not possible to distinguish between hypotheses favoring retention or independent loss of the same IR region in cupressophyte and Pinaceae cp genomes.

The present study focuses on establishing the complete cp genome sequence of a further member of the Podocarpaceae family, the Brazilian endemic species *P. lambertii*. Here, we characterize the cp genome organization of *P. lambertii* and compare its cp genome structure with other conifer species.

## Materials and Methods

### Plant material and cp DNA purification

Chloroplast isolation of *P. lambertii* was performed from young plants collected at a private area located at Lages, Santa Catarina, Brazil (27° 48′ 57" S, 50° 19′ 33" W), where the species is abundant, with previous permission from the owner (José Antônio Ribas Ribeiro). This species is not considered threatened. Afterwards, the young plants were transplanted to the greenhouse until the collection of needles. The cpDNA isolation was performed according to Vieira et al. [24].

### Chloroplast genome sequencing, assembling and annotation

Approximately 50 ng of cp DNA were used to prepare sequencing libraries with Nextera DNA Sample Prep Kit (Illumina Inc., San Diego, CA) according to the manufacturer's instructions. Chloroplast DNA was sequenced using Illumina MiSeq (Illumina Inc., San Diego, CA) at the Federal University of Paraná, Brazil. In total, 495,071 paired-end reads (2×250 bp) were obtained, and *de novo* assembly was performed using Newbler 2.6 v. The obtained paired-end reads were mapped on *P. lambertii* cp genome and the genome coverage estimated using the CLC Genomics Workbench 5.5 software. By using this approach, a total of 377,437 paired-end reads (76.23%) was obtained from cpDNA, resulting in 1,200-fold genome coverage. Initial annotation of the *P. lambertii* cp genome was performed using Dual Organellar GenoMe Annotator (DOGMA) [25]. From this initial annotation, putative starts, stops, and intron positions were determined based on comparisons to homologous genes in other cp genomes. The tRNA genes were further verified by using tRNAscan-SE [26]. A physical map of the cp circular genome was drawn using OrganellarGenomeDRAW (OGDRAW) [27]. The complete nucleotide sequence of *P. lambertii* cp genome was deposited in the GenBank database under accession number KJ010812.

### Comparative analysis of genome structure

We used the PROtein MUMmer (PROmer) Perl script in MUMmer 3.0 [28], available at http://mummer.sourceforge.net/, to visualize gene order conservation (dot-plot analyses) between *P. lambertii* and the non-Pinaceae conifer representatives *P. totara* (Podocarpaceae), *Cephalotaxus oliveri, Cephalotaxus wilsoniana* (Cephalotaxaceae), *Taxus mairei* (Taxaceae), *Taiwania cryptomerioides*, *T. flousiana* (Cupressaceae), *C. japonica* (Cupressaceae), as well as *Pinus thunbergii*, a Pinaceae representative.

### Repeat sequence analysis and IR identification

Simple sequence repeats (SSRs) were detected using MISA perl script, available at (http://pgrc.ipk-gatersleben.de/misa/), with thresholds of eight repeat units for mononucleotide SSRs, four repeat units for di- and trinucleotide SSRs, and three repeat units for tetra-, penta- and hexanucleotide SSRs. Tandem repeats were analyzed using Tandem Repeats Finder (TRF) [29] with parameter settings of 2, 7 and 7 for match, mismatch, and indel, respectively. The minimum alignment score and maximum period size were set as 50 and 500, respectively. All of the repeats found were manually verified, and the nested or redundant results were removed. REPuter [30] was used to visualize the remaining IRs in *P. lambertii* by forward *vs*. reverse complement (palindromic) alignment. The minimal repeat size was set to 30 bp and the identity of repeats ≥90%.

## Results and Discussion

### Chloroplast genome sequencing, assembling and annotation

**Figure 1:**
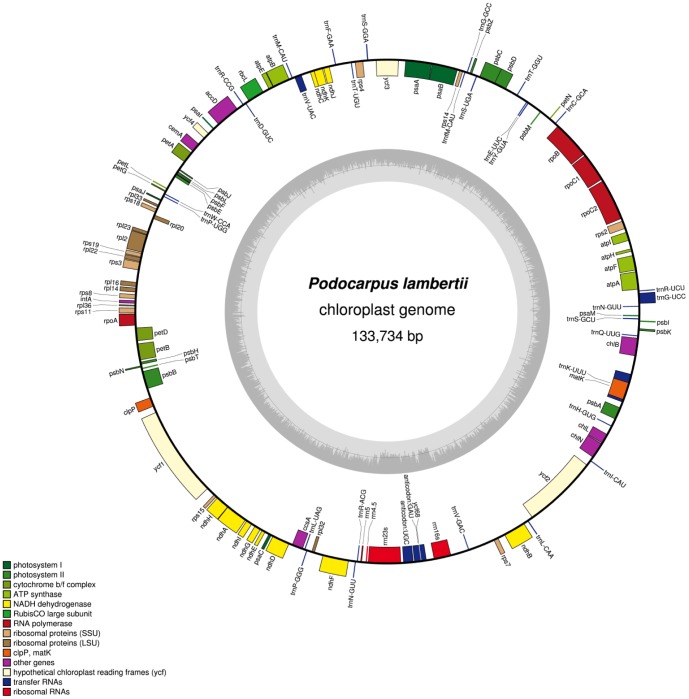
Gene map of *Podocarpus lambertii* chloroplast genome. Genes drawn inside the circle are transcribed clockwise, and genes drawn outside are counterclockwise. Genes belonging to different functional groups are color-coded. The darker gray in the inner circle corresponds to GC content, and the lighter gray corresponds to AT content.

*P. lambertii* cp genome size was determined to be 133,734 bp, which is very similar to *P. totara* (133,259 bp) (NC_020361.1) and larger than the sequenced cp genomes of Pinaceae species, which range from 116,479 bp in *Pinus monophylla* [14] to 124,168 bp in *Picea morrisonicola* [31]. *P. lambertii* cp genome size is smaller than the cp sequences in the cycads *Cycas taitungensis* (163,403 bp) [32] and *Cycas Revoluta* (162,489 bp) (NC_020319.1). The genome size of *P. lambertii* cp is consistent with the size of non-Pinaceae conifer species, which ranges from 127,665 bp in *T. mairei* (NC_020321.1) to 136,196 bp in *C. wilsoniana* [20]. A total of 119 genes were identified in the *P. lambertii* cp genome, of which 118 genes were single copy and one gene, *trnN*-GUU, was duplicated and occurred as an inverted repeat sequence. The following genes were identified and are listed in Figure 1 and Table 1: 4 ribosomal RNA genes, 31 unique transfer RNA genes, 20 genes encoding large and small ribosomal subunits, 1 translational initiation factor, 4 genes encoding DNA-dependent RNA polymerases, 50 genes encoding photosynthesis-related proteins, 8 genes encoding other proteins, including the unknown function gene *ycf2*, and 1 pseudogene, *ycf68*. Among these 118 single copy genes, 14 were genes containing introns (Table 1). The GC content determined for *P. lambertii* cp genome is 37.1%, which is higher than *C. oliveri* (35.2%), *C. wilsoniana* (35.1%), *T. cryptomerioides* (34.6%), and *C. japonica* (35.4%), but lower than *C. taitungensis* (39.5%) and *P. thunbergii* (38.8%).

**Table 1:** List of genes identified in *Podocarpus lambertii* chloroplast genome.

Category of Genes	Group of gene			Name of gene			
**Self-replication**	Ribosomal RNA genes	*rrn16*	*rrn23*	*rrn5*	*rrn4.5*		
	Transfer RNA genes	*trnA*-UGC*	*trnC*-GCA	*trnD*-GUC	*trnE*-UUC	*trnF*-GAA	*trnfM*-CAU
		*trnG*-UCC*	*trnG*-GCC	*trnH*-GUG	*trnI*-CAU	*trnI*-GAU*	*trnK*-UUU*
		*trnL*-CAA	*trnL*-UAG	*trnM*-CAU	*trnN*-GUU**	*trnP*-GGG	*trnP*-UGG
		*trnQ*-UUG	*trnR*-ACG	*trnR*-UCU	*trnR*-CCG	*trnS*-GCU	*trnS*-UGA
		*trnS*-GGA	*trnT*-UGU	*trnT*-GGU	*trnV*-GAC	*trnV*-UAC*	*trnW*-CCA
		*trnY*-GUA					
	Small subunit of ribosome	*rps2*	*rps3*	*rps4*	*rps7*	*rps8*	*rps11*
		*rps12* *	*rps14*	*rps15*	*rps18*	*rps19*	
	Large subunit of ribosome	*rpl2* *	*rpl14*	*rpl16*	*rpl20*	*rpl22*	*rpl23*
		*rpl32*	*rpl33*	*rpl36*			
	DNA-dependent RNA polymerase	*rpoA*	*rpoB*	*rpoC1* *	*rpoC2*		
	Translational initiation factor	*infA*					
**Genes for photosynthesis**	Subunits of photosystem I	*psaA*	*psaB*	*psaC*	*psaI*	*psaJ*	*psaM*
		*ycf3* *	*ycf4*				
	Subunits of photosystem II	*psbA*	*psbB*	*psbC*	*psbD*	*psbE*	*psbF*
		*psbH*	*psbI*	*psbJ*	*psbK*	*psbL*	*psbM*
		*psbN*	*psbT*	*psbZ*			
	Subunits of cytochrome	*petA*	*petB* *	*petD* *	*petG*	*petL*	*petN*
	Subunits of ATP synthase	*atpA*	*atpB*	*atpE*	*atpF* *	*atpH*	*atpI*
	Large subunit of Rubisco	*rbcL*					
	Chlorophyll biosynthesis	*chlB*	*chlL*	*chlN*			
	Subunits of NADH dehydrogenase	*ndhA* *	*ndhB* *	*ndhC*	*ndhD*	*ndhE*	*ndhF*
		*ndhG*	*ndhH*	*ndhI*	*ndhJ*	*ndhK*	
**Other genes**	Maturase	*matK*					
	Envelope membrane protein	*cemA*					
	Subunit of acetyl-CoA	*accD*					
	C-type cytochrome synthesis gene	*ccsA*					
	Protease	*clpP*					
	Component of TIC complex	*ycf1*					
**Genes of unknown function**	Conserved open reading frames	*ycf2*					
**Pseudogenes**		*ycf68*					

*Genes containing introns.

**Duplicated gene.

### Gene content differences

The gene content of *P. lambertii* cp genome and that of other conifer cp genomes sequenced to date show high similarity. However, some differences are observed when we compare *P. lambertii* cpDNA with other non-Pinaceae and Pinaceae conifers. One exception is the *rps16* gene, which is absent from the *P. lambertii* cp genome. This result reinforce the apparently loss of rps16 gene in Podocarpaceae and Araucariaceae families. Wu and Chaw [16] reported the *rps16* gene loss in *Nageia nagi* (Podocarpaceae) and *Agathis dammara* (Araucariaceae). This gene is present in other non-Pinaceae conifer cp genomes published so far [11], [12], [20], [32]. The *rps16* gene loss has already been reported in other gymnosperms, such as Pinaceae and Gnetophyte species [23], [32], [33]. Wu et al. [20] considered *rps16* gene loss as a structural mutation unique to the cpDNAs of gnetophytes and Pinaceae, but since the loss of this gene has been identified in Podocarpaceae and Araucariaceae families, we can consider that some cupressophytes may also present this mutation. This gene is also absent, or nonfunctional, in some angiosperm species of the Fabaceae family, such as *Medicago truncatula*, in which it is completely absent, and in *Phaseolus vulgaris* and *Vigna radiata*, in which it is nonfunctional. In this angiosperm family, the coding sequence contains many internal stop codons and a modified initial stop codon [34], [35]. Since this gene was shown to be essential for cell survival in tobacco [36], it was probably transferred to the nucleus, as observed for different species of the Fabaceae family [34], [35], and has since become a functional nuclear gene required for normal plastid translation.

The *trnP*-GGG and *trnR*-CCG genes are considered to be relics of plastid genome evolution in gymnosperms, pteridophytes and bryophytes [37]. The *trnP*-GGG gene is present in the *P. lambertii* cp genome, as well as such conifer species as *C. japonica, P. thunbergii*, *C. oliveri* and *C. wilsoniana* and other gymnosperm species, such as *C. taitungensis*, *Gnetum* and *Ginkgo*. The *trnR*-CCG gene is present as complete and functional tRNA in *P. lambertii* (Podocarpaceae), as well as the cp genomes of *P. thunbergii* (Pinaceae), *C. taitungensis* (Cycadaceae) [32], whereas it is absent from *C. japonica* (Cupressaceae), *C. oliveri* and *C. wilsoniana* (Cephalotaxaceae), and *T. mairei* (Taxaceae) [11], [12]. Hirao et al. [12] suggested that *trnR*-CCG might have been completely lost in the Cupressaceae *s.l.*, which has only relatively recently diverged during the long evolutionary history of plants. These data corroborate the hypothesis based on phytochrome phylogenetic trees, in which the most ancient branch of the conifers seems to be the Pinaceae, and the next split appears to have separated Araucariaceae plus Podocarpaceae from the Taxaceae/Taxodiaceae/Cupressaceae group [38]. This trnR-CCG gene may have been lost during the second split separating Araucariaceae and Podocarpaceae taxa. In addition, *trnT*-GGU occurs as a pseudogene in the *C. japonica* cp genome, with only 43 bp, while it is present and completely functional in *P. lambertii* and *C. oliveri*, *C. wilsoniana*, duplicated in *P. thunbergii*, and totally absent from the *C. taitungensis* cp genome. Interestingly, the *trnT*-GGU gene is highly conserved in angiosperms, and knockout of this gene in tobacco plants produced viable plants, whereas the growth of these plants was strongly affected, suggesting an important role during plastid translation [39]. The loss of the *trnT*-GGU gene in several gymnosperm species suggests that a uridine modification in the anticodon position of the *trnT*-UGU gene occurred during evolution, which would facilitate the reading of threonine codons and makes the *trnT*-GGU gene dispensable in these species [39]-[42]. Evolutionarily, the loss of this tRNA gene could be used as a tool, or marker gene, to study the possible ways that the conifers diverged during evolution. However, it remains to be determined whether structural differences in the cp ribosome or modification in the structure of this tRNA, between angiosperms and gymnosperms, would facilitate the decoding.

### Comparative analysis of genome structure

Chloroplast genome organization is much conserved in angiosperms, as well as the presence of IRs, with very few exceptions. As reported by Terakami et al. [43] in *Pyrus*, *Malus* and *Nicotiana*, neither translocation nor inversion was detected in the three species. In addition, considering the many dicot and monocot species, only one large inversion was reported [43].

**Figure 2:**
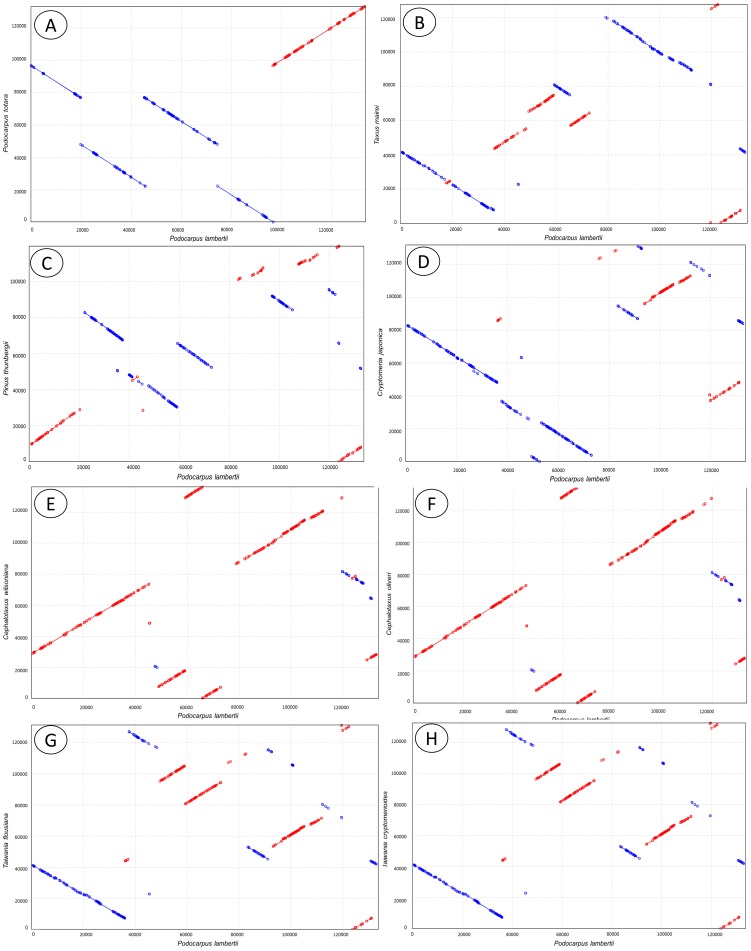
Dot-plot analyses of eight sampled conifer chloroplast DNAs against *Podocarpus lambertii*. A positive slope denotes that the two compared sequences are in the same orientation, whereas a negative slope indicates that the compared sequences can be aligned, but their orientations are opposite. Graphs represents comparisons between *Podocarpus lambertii* (axis X) and *Podocarpus totara* (A), *Taxus mairei* (B), *Pinus thunbergii* (C), *Cryptomeria japonica* (D), *Cephalotaxus wilsoniana* (E), *Cephalotaxus oliveri* (F), *Taiwania flousiana* (G), and *Taiwania cryptomerioides* (H) in axis Y.

In addition to the loss of the large IR in conifers, many genome rearrangements were observed in the cp genome, and such rearrangements appear to play an important role in their evolution. Dot-plot analyses indicate that the structure of the *P. lambertii* cp genome differs significantly from cp genomes of other conifer species, and, surprisingly, it has significant differences when compared to *P. totara* (Figure 2A-H).

For the genus *Cephalotaxus s.l.*, specifically *C. wilsoniana* and *C. Oliveri*, it was shown that the genome structures were almost the same [11]. Similar results were observed in the present study, as revealed by the high similarity in the dot-plot analyses between *Podocarpus* and *Cephalotaxus* genera, as represented by *P. lambertii* × *C. wilsoniana* (Figure 2E) and *P. Lambertii* × *C. oliveri* (Figure 2F), and between the *Podocarpus* and *Taiwania* genera, as represented by *P. lambertii* × *T. flousiana* (Figure 2G) and *P. lambertii* × *T. cryptomerioides* (Figure 2H). This high similarity in dot-plot analysis indicates the occurrence of exactly the same structural modifications between *P. lambertii* and these two *Cephalotaxus* and *Taiwania species*.

Differently, for *P. lambertii* and *P. totara* (Figure 2A), we observed four large inversions of about 20,000 bp in length each. In both *Cephalotaxus* and *Taiwania* genera, the two sequenced species share the same region of natural occurrence, which is not true for either *Podocarpus* species sequenced. Thus, these large inversions can be explained by, and probably result from, the large distance between the natural occurrence of these two species in that *P. lambertii* occurs in Brazil, while *P. totara* occurs in New Zealand. Moreover, podocarps have a rich fossil record that suggests an origin in the Triassic period (about 220 million years) and a distribution in both the Northern and Southern Hemispheres through the Cretaceous and earliest Tertiary periods, about 100 million years ago [44]–[46]. Thus, geographic distance and different adaptive traits could explain the structural differences found between these two species of the same genera.

**Figure 3:**
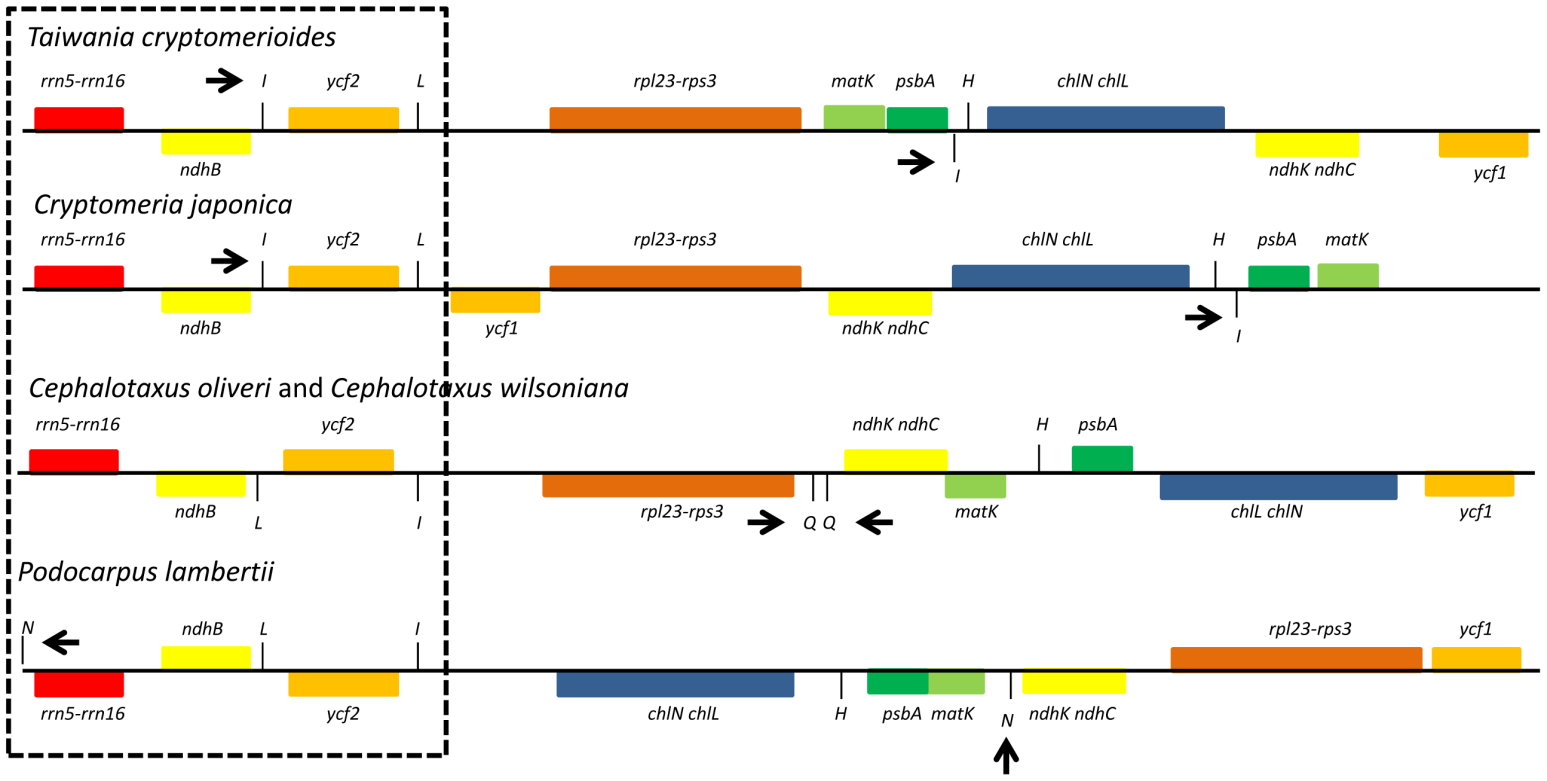
Comparison of IR and genome structure in 5 cupressophytes. Five cupressophyte species from top to bottom are *Taiwania cryptomerioides*, *Cryptomeria japonica*, *Cephalotaxus oliveri*, *Cephalotaxus wilsoniana* and *Podocarpus lambertii*. Genes are represented by boxes extending above or below the baseline, according to the direction of transcription; genes with the same function have the same color. Transfer RNA genes are abbreviated as the type of one letter. Dashed boxes represent the retained IR region, and arrows indicate the short IR on each species. Adapted from Yi et al. (2013).

In addition, the loss of one large IR copy already reported in other conifer species were also observed in the *P. lambertii* cp genome [11], [12], [20]. However, short remaining IR sequences of 326 bp can be found in *P. lambertii*, 544 bp in *C. oliveri*, 530 bp in *C. wilsoniana*, 277 bp in *T. cryptomerioides* and 284 bp in *C. japonica* [11]. These short remaining IR sequences also differ in the nucleic acid sequences and gene content between different conifer species. In *P. lambertii*, *trnN*-GUU remain from the lost IR copy region, while in *T. cryptomerioides* and *C. japonica*, *trnI*-CAU remained after the rearrangements that determined the loss of one IR copy [11]. In *C. oliveri* and *C. wilsoniana*, the *trnQ*-UUG is duplicated; however, this gene is not normally present in the IR region, and its duplication was probably produced by other rearrangements not involved with the IR regions [20]. After much evidence provided by different conifer plastid genomes, it can be concluded that the loss of one IR copy occurred after a reduction in sequence and gene content and that such loss was most likely caused by this reduction [11], [12], [14], [20], [23], [32], [33]. However, this speculation remains to be established. To date, it is not entirely clear whether cupressophytes and Pinaceae species have lost different IR regions [11]. However, we can observe in *P. lambertii* an inversion in the direction of transcription of ribosomal RNA genes spanning *rrn5*-*rrn16* and protein-coding genes, *ndhB* and *ycf2*, when compared to *C. oliveri*, *C. wilsoniana, T. cryptomerioides* and *C. japonica* (Figure 3).

### Repeat sequence analysis

The cp genome mode of inheritance, paternal in most gymnosperms, allows us to elucidate the relative contributions of seed and pollen flow to the genetic structure of natural populations by comparison of nuclear and cp markers [47]. The cp microsatellites, or SSRs, may be identified in completely sequenced plant cp genomes by simple database searches, followed by primers designed to screen for polymorphism. To date, studies of cp microsatellites have revealed much higher levels of diversity than have those of cp restriction fragment length polymorphisms (RFLP) [47]–[49].

We have analyzed the occurrence, type, and distribution of SRRs in the *P. lambertii* cp genome. In total, 156 SSRs were identified. Among them, homo- and dipolymers were the most common with, respectively, 80 and 63 occurrences, whereas tri- (4), tetra- (7), penta- (1), and hexapolymers (1) occur with lower frequency (Table 2). Most homopolymers are constituted by A/T sequences (87.5%), and of the dipolymers, 61.1% were also constituted by multiple A and T bases.In this study, we identified 78 repeats with more than one nucleotide repeat, totaling almost 50% of all SSRs identified. The 13 tri-, tetra-, penta-, and hexapolymers are shown in Table 3, as well as their size and location. From these 13 polymers identified, 9 are localized in intergenic spacers, 3 in coding sequences, and only 1 inside an intron. These results reveal the presence of several SSR sites in *P. lambertii*. Hereafter, these sites can be assessed for the intraspecific level of polymorphism, leading to highly sensitive phylogeographic and population structure studies for this species.

**Table 2:** List of simple sequence repeats identified in *Podocarpus lambertii* chloroplast genome.

SSR sequence	Number of repeats													TOTAL
	**3**	**4**	**5**	**6**	**7**	**8**	**9**	**10**	**11**	**12**	**13**	**14**	**15**	
A/T	–	–	–	–	–	39	14	6	4	6	–	–	1	70
C/G	–	–	–	–	–	–	3	3	–	1	1	–	2	10
AC/GT	–	1	–	1	–	–	–	–	–	–	–	–	–	2
AG/CT	–	21	1	–	–	–	–	–	–	–	–	–	–	22
AT/AT	–	24	7	2	2	–	3	1	–	–	–	–	–	39
AAG/CTT	–	1	–	–	–	–	–	–	–	–	–	–	–	1
AAT/ATT	–	3	–	–	–	–	–	–	–	–	–	–	–	3
AATC/ATTG	2	–	–	–	–	–	–	–	–	–	–	–	–	2
AATG/ATTC	1	–	–	–	–	–	–	–	–	–	–	–	–	1
AATT/AATT	1	–	–	–	–	–	–	–	–	–	–	–	–	1
ACAT/ATGT	1	–	–	–	–	–	–	–	–	–	–	–	–	1
ACCT/AGGT	1	–	–	–	–	–	–	–	–	–	–	–	–	1
AGAT/ATCT	3	–	–	–	–	–	–	–	–	–	–	–	–	3
AAATG/ATTTC	1	–	–	–	–	–	–	–	–	–	–	–	–	1
AGATAT/ATATCT	1	–	–	–	–	–	–	–	–	–	–	–	–	1
**TOTAL**														**158**

**Table 3:** Distribution of tri-, tetra-, penta-, and hexapolymer simple sequence repeats (SSRs) loci in *Podocarpus lambertii* chloroplast genome.

SSR type	SSR sequence	Size	Start	End	Location
penta	(AATGA)3	15	21884	21898	*trnE*-*UUC/trnT*-GGU (IGS)
hexa	(AGATAT)3	18	37894	37911	*trnF*-GAA/*ndhJ* (IGS)
tetra	(ATCA)3	12	44346	44357	*atpE*/*rbcL* (IGS)
tri	(AAG)4	12	75761	75772	*Ycf1* (CDS)
tetra	(AATG)3	12	86350	86361	*ndhA* (intron)
tetra	(TGAT)3	12	97140	97151	*ndhF*/*trnN*-GUU (IGS)
tetra	(CTAC)3	12	99809	99820	*rrn23* (CDS)
tri	(ATT)4	12	103664	103675	*trnI*-GAU/*rrn16* (IGS)
tri	(ATA)4	12	120539	120550	*rps7*/*ndhB* (IGS)
tri	(TTA)4	12	122046	122057	*chlL* (CDS)
tetra	(AATT)3	12	122977	122988	*chlL*/*trnH*-GUG (IGS)
tetra	(CATA)3	12	125437	125448	*psbA*/*trnK*-UUU (IGS)
tetra	(ATAG)3	12	125570	125581	*psbA*/*trnK*-UUU (IGS)

CDS, coding sequences; IGS, intergenic spacers.

Tandem repeats with more than 30 bp and with a sequence identity of more than 90% have also been examined. Twenty-eight tandem repeats were identified in the *P. lambertii* cp genome (Table 4), of which 15 are located in coding regions of *accD* (2), *rps18* (1), *rps19* (1), *rps11* (1), *ycf1* (8), *rpl32* (1), *ycf2* (1); 11 are distributed in the intergenic spacers of *atpA*/*atpF* (1), *trnR*-CCG/*accD* (1), *rpl2*/*rps19* (1), *clpP*/*ycf1* (2), *ndhE*/*psaC* (1), *trnR*-ACG/*rrn5* (1), *rps12*/*rps7* (1), *ycf2*/*trnI*-CAU (1), *trnQ*-UUG/*psbK* (1), *psbK*/*psbI* (1); and 2 are located in the intron sequence of *rpoC1*. The cp genome of *P. lambertii* has 11 tandem repeats, more than the cp genome of *C. oliveri*, as well as a higher number of repeats in the *ycf1* (6) gene coding sequence [11]. The *ycf1* gene, previously considered as an enigmatic function in the cp genome, has recently been identified as encoding an essential protein component of the cp translocon at the inner envelope membrane (TIC) [50]. In *Salvia miltiorrhiza* and *Cocos nucifera*, two angiosperms, only 7 and 8 tandem repeats, respectively, of about 20 bp were identified, none of them located at the *ycf1* coding sequence [51], [52], corroborating the theory that the IR influences the stability of the plastid genome.

**Table 4:** Distribution of tandem repeats in *Podocarpus lambertii* chloroplast genome.

Serial Number	Repeat Length (bp)	Consensus size × Copy number	Start-End	Location
1	32	16×2	3450-3482	*atpA*/*atpF* (IGS)
2	284	142×2	13170–13454	*rpoC1* (Intron)
3	60	30×2	13496–13557	*rpoC1* (Intron)
4	30	15×2	46625–46653	*trnR*-CCG/*accD* (IGS)
5	90	30×3	47533–47619	*accD* (CDS)
6	42	21×2	48149–48192	*accD* (CDS)
7	52	26×2	57988–58043	*rps18* (CDS)
8	32	16×2	61875–61905	*rpl2*/*rps19* (IGS)
9	54	18×3	62177–62237	*rps19* (CDS)
10	63	21×3	66568–66630	*rps11* (CDS)
11	32	16×2	75172–75203	*clpP*/*ycf1* (IGS)
12	104	52×2	75412–75529	*clpP*/*ycf1* (IGS)
13	36	18×2	79255–79292	*ycf1* (CDS)
14	162	52×3	79351–79504	*ycf1* (CDS)
15	162	81×2	79362–79519	*ycf1* (CDS)
16	108	27×4	79401–79519	*ycf1* (CDS)
17	132	33×4	80478–80619	*ycf1* (CDS)
18	96	24×4	80732–80820	*ycf1* (CDS)
19	273	21×13	81305–81571	*ycf1* (CDS)
20	96	48×2	82408–82528	*ycf1* (CDS)
21	30	15×2	89787–89817	*ndhE*/*psaC* (IGS)
22	126	42×3	93843–93963	*rpl32* (CDS)
23	64	32×2	97838–97902	*trnR*-ACG/*rrn5* (IGS)
24	300	60×5	109209–109531	*rps12*/*rps7* (IGS)
25	36	12×3	116515–116547	*ycf2* (CDS)
26	60	20×3	119998–120055	*ycf2*/*trnI*-CAU (IGS)
27	128	64×2	131733–131853	*trnQ*-UUG/*psbK* (IGS)
28	26	13×2	132530–132556	*psbK*/*psbI* (IGS)

CDS, coding sequences; IGS, intergenic spacers.

Yi et al. [11] attributed the expansion of the *accD* ORF to the presence of tandemly repeated sequences. In the *P. lambertii* cp genome, we identified 2 tandem repeats in *accD* CDS, totaling 132 bp, or 44 codons. The *accD* reading frame length of the *P. lambertii* cp genome is 864 codons, similar to other cupressophyte species, such as *C. oliveri* (936 codons), *C. wilsoniana* (1,056 codons), *C. japonica* (700 codons) and *T. cryptomerioides* (800 codons). In contrast, the reading frame lengths of cycads, Ginkgo and Pinaceae, range from 320 to 359 codons, less than half the size found in cupressophytes. These results support the hypothesis of Hirao et al. [12] and Yi et al. [11] which holds that the *accD* reading frame has displayed a tendency toward enlarging sizes in cupressophytes.

The complete cp genome sequence of *P. lambertii* revealed significant structural changes occurring in the cp genome, even in species from the same genus. These results reinforce the apparently loss of rps16 gene in Podocarpaceae cp genome. In addition, several SSRs in the *P. lambertii* cp genome are likely intraspecific polymorphism sites which may allow highly sensitive phylogeographic and population structure studies, as well as phylogenetic studies, of species of this genus.
